# Association between remnant cholesterol and verbal learning and memory function in the elderly in the US

**DOI:** 10.1186/s12944-022-01729-4

**Published:** 2022-11-14

**Authors:** Ying-Yi Xie, Liang Zhao, Li-Jian Gao, Rui-Xia Xu, Ying Gao, Ke-Fei Dou, Yuan-Lin Guo, Yong-Ming He

**Affiliations:** 1 Cardio-Metabolic Medicine Center, National Center for Cardiovascular Diseases, Fuwai Hospital, Chinese Academy of Medical Sciences, Peking Union Medical College, Beijing, China; 2 Coronary Heart Disease Center, National Center for Cardiovascular Diseases, Fuwai Hospital, Chinese Academy of Medical Sciences, Peking Union Medical College, Beijing, China; 3 Division of Cardiology, The First Affiliated Hospital of Soochow University, Suzhou, Jiangsu Province China

**Keywords:** Remnant cholesterol, Total cholesterol/remnant cholesterol, Cognition, Verbal learning and memory function, Elderly, Cross-sectional study

## Abstract

**Background:**

The relationship between remnant cholesterol (RC) and atherosclerotic cardiovascular risk has been given increasing attention in recent years. However, its association with verbal learning and memory performance has not been reported.

**Methods:**

Data were extracted from the National Health and Nutrition Examination Survey (NHANES) 2011–2014 database. Participants aged ≥60 years with available fasting lipid data were included. Verbal learning and memory performance were evaluated using the **C**onsortium to **E**stablish a **R**egistry for **A**lzheimer’s **D**isease **W**ord **L**ist Memory Task (CERAD-WL) subtest. The CERAD total score was calculated as the mean of three immediate recalls and a delayed recall. RC was calculated as total cholesterol (TC) minus the sum of low-density lipoprotein cholesterol (LDL-C) and high-density lipoprotein cholesterol (HDL-C). Multivariate ordinal logistic regression was performed to evaluate the association between RC, as well as its derived marker, the TC/RC ratio, and age-stratified quartiles of the CERAD total score.

**Results:**

A total of 1377 participants were analysed. On a continuous scale, per 1 mmol/L increase in RC and per 1 unit increase in the TC/RC ratio were associated with multivariable adjusted odds ratios (95% CI) of 0.74 (0.58–0.94) and 1.45 (1.13–1.87), respectively, for having a CERAD total score in a higher quartile. On a categorical scale, higher RC quartiles were associated with a CERAD total score in a lower quartile; in contrast, the higher TC/RC quartile was associated with a CERAD total score in a higher quartile (all *P for trend* < 0.05).

**Conclusions:**

The current study suggests that lower RC levels and a higher TC/RC ratio are associated with better verbal learning and memory function, which indicates that lowering RC levels could be beneficial for preventing cognitive impairment in elderly individuals. Further research is needed to validate the causal roles of RC and the TC/RC ratio in cognition.

**Supplementary Information:**

The online version contains supplementary material available at 10.1186/s12944-022-01729-4.

## Introduction

Dementia is a vital health problem in the global growing aging population. The estimated dementia population will increase from 57.4 (50.4–65.1) million cases globally in 2019 to 152.8 (130.8–175.9) million cases in 2050 [1]. Given the lack of effective pharmacological therapies for dementia thus far, the primary prevention of modifiable risk factors has become crucial in addressing the rising epidemic of cognitive impairment.

Dyslipidemia has been found to be independently associated with cognitive impairment. In observational studies, higher serum TC levels in mid-life were associated with a higher risk of developing dementia in late life [2], but the impact of serum TC levels in late life on the development of dementia remains unclear. A meta-analysis synthesizing 34 cohort studies found no association between serum TC levels and mild cognitive impairment, Alzheimer’s disease (AD), vascular dementia, any dementia, or cognitive decline [3]. Studies on serum LDL-C levels and dementia or cognitive decline also displayed differential results and seemed to vary by sex and the presence of cardiovascular risk factors [4]. However, a Mendelian randomization study showed that genetically low serum levels of LDL-C reduced the risk of AD [5]. Overall, inconsistent results have been found in the association between serum lipid profiles and cognitive impairment, limiting the clinical utility of lipid biomarkers. In addition to further research on traditional blood lipid parameters, the exploration of new lipid parameters to predict cognitive function is urgently needed, especially in the elderly population.

Remnant cholesterol (RC) represents the amount of cholesterol in the remnant lipoproteins transformed from triglyceride-rich lipoproteins in the blood [6]. Recently, RC has been shown to equally or even more precisely predict cardio-cerebrovascular outcomes compared to LDL-C or HDL-C [7–9]. Elevated RC levels are closely related to triglyceride metabolism disorders and insufficient APOE-mediated remnant lipoprotein clearance by the liver, such as type III hyperlipidemia [10]. Meanwhile, *APOE* variants are closely related to the risk of AD. Therefore, RC has the biological possibility of affecting cognitive function. However, no study has reported whether or how RC is associated with cognitive function.

In seeking a more plausible and reliable lipid marker of late-life cognitive impairment, the present study explored the relationship of RC with cognitive function utilizing data from a cohort of American elderly individuals in the National Health and Nutrition Examination Survey (NHANES) conducted between 2011 and 2014. Given the complexity of the metabolism of remnant lipoprotein in human plasma, the study also explored a new lipid marker derived from RC, the total cholesterol to remnant cholesterol (TC/RC) ratio, to further investigate the relationship of RC with cognitive function.

## Methods

### Study design and population

The NHANES database is a cross-sectional program with a 2-year-cycle design that aims to assess the health and nutritional status of civilian, noninstitutionalized resident populations in the United States. A complex, multistage sampling procedure was implemented to select eligible representative participants. The present study made use of the 2011–2012 and 2013–2014 cycles with 19,931 individuals, of which participants aged ≥60 years were eligible for cognitive function assessment and enrolled for analysis. Participants without fasting plasma lipid data or complete cognitive assessment results were excluded. Those without complete measurements of TC, LDL-C, and HDL-C values for the calculation of RC were also excluded.

The NHANES protocols were approved by the National Center for Health Statistics Ethics Review Board of the U.S. CDC. All participants received and completed written informed consent during the survey. All material for analysis can be accessed on the NHANES official website [11].

### Measurement of blood lipids and the definition of exposure variables

TC and triglycerides (TGs) were measured using an enzymatic assay method. HDL-C was measured using the heparin-manganese precipitation method or a direct immunoassay technique. LDL-C was calculated from measured values of TC, TGs, and HDL-C according to the Friedewald formula as follows: [LDL-C] = [TC] – [HDL-C] – [TG/5]. The formula is valid for TG values less than or equal to 400 mg/dL. The exposure variables were remnant cholesterol (RC) and its derivative, the TC/RC ratio. RC was calculated as TC minus the sum of LDL-C and HDL-C [RC = TC-(LDL-C + HDL-C)], and the TC/RC ratio was calculated as TC divided by RC.

### Assessment of verbal learning and memory function and the definition of the outcome variable

In NHANES 2011–2014, a series of evaluations of cognitive functioning were conducted during the MEC survey, including the word learning and recall modules from the Consortium to Establish a Registry for Alzheimer’s Disease (CERAD W-L). The CERAD W-L assesses the immediate and delayed learning ability for new verbal information (memory subdomain), consisting of three sequential learning trials and a delayed recall trial. For learning trials, participants were tasked with reading 10 unrelated words displayed on a screen aloud, one at a time, and were asked to recall as many words as possible immediately after their presentation. A delayed recall test was administered after the other two cognitive tests were completed. The outcome variable was the CERAD total score, which was calculated as the average of all three immediate recalls after each learning trial and the delayed recall. Given the significant effect of age on cognitive function, the age- stratified (≥60 to 70, ≥70 to 80, and ≥80 years) quartiles of the CERAD total test score were used [12, 13] to evaluate verbal learning and memory function. Specifically, the age-stratified quartiles of the CERAD total test score were calculated by first dividing the studypopulation into 3 age groups (≥60 to 70, ≥70 to 80, and ≥80 years) and then categorizing participants into 4 grades of verbal learning and memory function (1 to 4) by the weighted quartiles of CERAD total score separately in the 3 age groups. The lowest quartile indicated impaired verbal learning and memory function. The higher the quartile, the better the learning and memory function.

### Covariates

Potential confounding factors were investigated, including age, sex (male and female), race, BMI status, educational level, smoking status, and drinking status. Race was defined as follows: 1) non-Hispanic white; 2) non-Hispanic black; and 3) other races, including Mexican American, other Hispanic, non-Hispanic Asian and multiracial. Educational level was defined as follows: 1) less than high school: less than 9^th^ grade or no high school diploma; 2) high school graduate: high school graduate/GED or equivalent; and 3) college or above: some college or AA degree, college graduate or above. BMI was categorized into 1) normal, 2) overweight and 3) obese using 25 kg/m^2^ and 30 kg/m^2^ as the cutoffs. Smoking status was defined as follows: 1) current smokers: had smoked more than 100 cigarettes (including hand rolled cigarettes, cigars, and cigarillos) in their lifetime and had smoked in the last 28 days; 2) ex-smokers: had smoked more than 100 cigarettes in their lifetime but had not smoked in the last 28 days; and 3) never smokers: had not smoked more than 100 cigarettes in their lifetime and did not currently smoke [14]. Drinking status was defined as follows: 1) current drinker: had drunk at least 12 drinks in their lifetime and had drunk at least 1 drink in the past year; 2) former drinker: had drunk at least 12 drinks in their lifetime and had drunk no drinks in past year; and 3) lifetime abstainer: had drunk fewer than 12 drinks in their lifetime [15].

Other variables included in the baseline characteristics and subgroup analysis were marital status, statin use, diabetes, and hypertension. Marital status was defined as 1) married/cohabitating and 2) not married/never married. Diabetes and hypertension were based on self-report questionnaire data.

### Statistical analyses

All statistical analyses were conducted by Stata SE 16.0 (Stata Corporation, College Station, TX). The complex survey design of the NHANES was taken into account by specifying primary sampling units (PSUs), strata, and sampling weights in the software’s *svyset* module. Sampling weights were constructed for the combined 2011–2012 and 2013–2014 4-year cycles by dividing the individual sampling weights by 2, according to the NHANES analytic tutorials [16].

Baseline characteristics were grouped by age-stratified quartiles of the CERAD total test score. Data are described as the mean (± standard deviation) for continuous variables, median (interquartile range) for skewed variables, and sample counts (weighted percentage) for categorical variables. A weighted trend test was performed across age-stratified quartile groups for each continuous and categorical variable.

In the statistical analysis of lipid-cognition associations, RC and the TC/RC ratio were treated as both continuous and categorical variables. Continuous RC and TC/RC variables were natural log-transformed to fit regression models due to their right-skewed distribution [7]. Categorical RC and TC/RC variables with four grades were created using quartiles as cutoffs. Multivariate ordinal logistic regression analysis was performed to evaluate the relationship among RC, the TC/RC ratio and grades of verbal learning and memory functioning. Models were further adjusted for potential risk factors or confounders based on prior studies. Model 1 included univariate analysis. Model 2 was adjusted for age, sex, race, and education level. Model 3 was further adjusted for BMI status, smoking status and drinking status plus the adjustments in Model 2. Subgroup analysis was performed to evaluate the robustness of the results in a diversity of demographic, disease-specific and lipid-stratified subgroups. A two-sided *P* < 0.05 was considered statistically significant.

## Results

### Characteristics of the study population

**Fig. 1:**
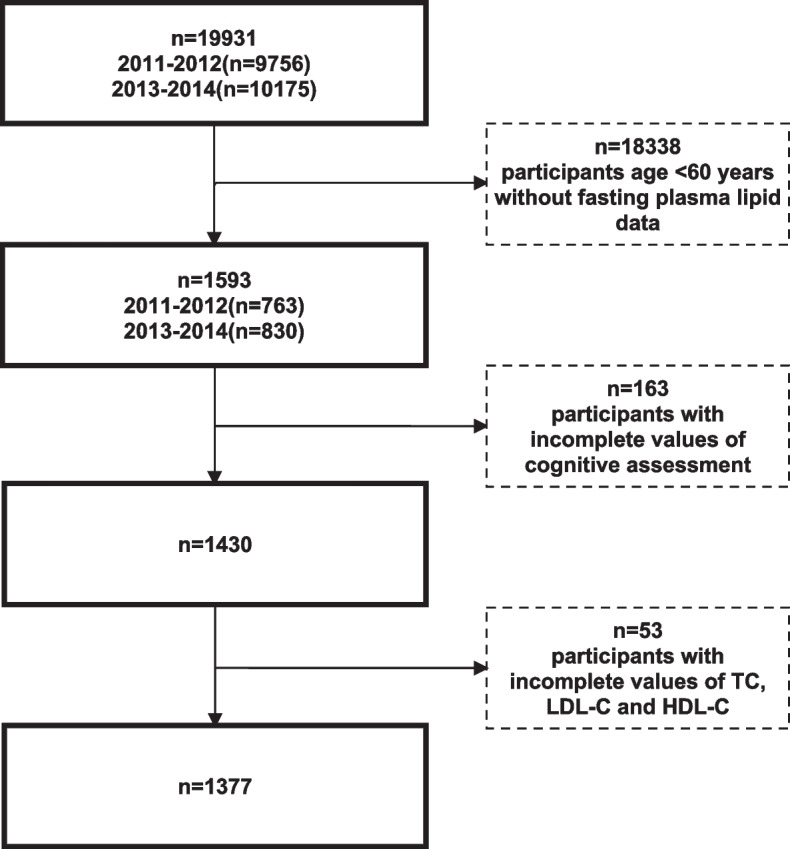
Flowchart of the sample selection process of the study population

Finally, according to the study population selection process, 1377 participants aged ≥60 years were included in the study (Fig. 1). The mean age of the participants was 69.1 ± 6.5 years, and 55% were male. A total of 79% of the participants were non-Hispanic white, 61% had an educational level of college or above, and 32% were not married or never married. Thirty-eight percent of the participants were obese (BMI ≥28 kg/m2), 19%had a history of diabetes, and 59% had hypertension. For the baseline lipid profile, the mean TC level was 4.97 ± 1.07 mmol/L, and the median RC level was 0.54 (0.39, 0.78) mmol/L. For CERAD test performance, 26% of the participants had scores in the lowest age-stratified quartile. Details are depicted in Table 1.

**Table 1:** Baseline characteristics of the study population grouped by age-stratified quartiles of the CERAD total score

		Groups by age-stratified quartiles of CERAD total score				
	Total (*n* = 1377)	1^st^ quartile (*n* = 458)	2^nd^ quartile (*n* = 381)	3^rd^ quartile (*n* = 305)	4^th^ quartile (*n* = 233)	*P for trend*
***Age, yrs***^***a***^	69.1 ± 6.5	69.6 ± 7.4	69.3 ± 6.3	68.8 ± 6.3	68.6 ± 6.9	< 0.001
***Gender, n(%)***^***c***^						
Female	705(55)	184(12)	197(14)	178(16)	146(14)	< 0.001
Male	672(45)	274(14)	184(13)	127(9.4)	87(7.5)	
**Race*, n(%)***^***c***^						
White	696(79)	180(18)	185(21)	171(21)	160(19)	< 0.001
Black	281(8.5)	110(3)	83(2.6)	57(1.8)	31(1)	
Other races	400(12)	168(4.8)	113(3.6)	77(2.4)	42(1.2)	
***Education level, n(%)***^***c***^						
Less than high school	356(16)	183(7.5)	110(5.4)	45(2.3)	18(1.1)	< 0.001
High school graduate	329(23)	113(6.8)	94(6.8)	72(5.5)	50(3.9)	
College or above	690(61)	161(11)	176(15)	188(17)	165(16)	
***Marital status, n(%)***^***c***^						
Married/Cohabiting	855(68)	294(18)	225(19)	190(17)	146(15)	< 0.001
Not married/Never married	522(32)	164(8)	156(9.2)	115(8.1)	87(6.3)	
***BMI, n(%)***^***c***^						
Normal	379(27)	122(6.3)	98(6.6)	88(7.2)	71(7.1)	< 0.001
Overweight	465(35)	155(9.3)	128(9.8)	100(8.4)	82(7.1)	
Obese	533(38)	181(10)	155(11)	117(9.4)	80(7.4)	
***Drinking status, n(%)***^***c***^						
Lifetime abstainer	215(14)	68(3.3)	69(4)	45(3.4)	33(2.8)	< 0.001
Former drinker	368(23)	136(7.4)	109(6.8)	77(5.2)	46(3.7)	
Current drinker	766(63)	239(15)	195(17)	180(16)	152(15)	
***Smoking status, n(%)***^***c***^						
Never smoker	682(49)	222(12)	179(13)	149(12)	132(12)	< 0.001
Former smoker	527(40)	173(10)	154(12)	122(11)	78(7.2)	
Current smoker	166(11)	63(3.7)	46(2.7)	34(2.5)	23(1.9)	
***Diabetes, n(%)***^***c***^	302(19)	108(6.3)	99(6.2)	61(3.7)	34(2.5)	< 0.001
***Hypertension, n(%)***^***c***^	859(59)	290(16)	241(17)	189(14)	139(12)	< 0.001
***Stroke, n(%)***^***c***^	99(6.7)	35(2)	34(2)	15(1.3)	15(1.4)	< 0.001
***Statin use, n(%)***^***c***^						
Statin user	850(60)	276(14)	223(16)	195(15)	156(15)	< 0.001
Non statin user	527(40)	182(12)	158(12)	110(9.6)	77(6.5)	
***TC, mmol/L***^***a***^	4.97 ± 1.07	4.77 ± 1.12	4.91 ± 1.02	5.08 ± 1.07	5.17 ± 0.99	< 0.001
***LDL-C, mmol/L ***^***a***^	2.87 ± 0.93	2.73 ± 1.00	2.81 ± 0.89	2.95 ± 0.91	3.02 ± 0.85	< 0.001
***HDL-C, mmol/L ***^***a***^	1.47 ± 0.43	1.38 ± 0.45	1.46 ± 0.38	1.54 ± 0.41	1.54 ± 0.42	< 0.001
***TG, mmol/L ***^***b***^	1.19 (0.85,1.71)	1.38 (0.90,1.75)	1.21 (0.89,1.75)	1.12 (0.78,1.64)	1.10 (0.81,1.68)	< 0.001
***RC, mmol/L ***^***b***^	0.54 (0.39,0.78)	0.63 (0.41,0.81)	0.55 (0.41,0.80)	0.51 (0.36,0.75)	0.50 (0.36,0.77)	< 0.001

^*^ Data were shown as ^a^mean (± standard deviation), ^b^median (interquartile range), ^c^unweighted counts (weighted percentage)

### Analysis of the association among RC, the TC/RC ratio and the CERAD total score

On a continuous scale, each 1 mmol/L increase in RC was associated with a multivariable adjusted odds ratio (95% CI) of 0.74 (0.58–0.94) for having a CERAD total score in a higher quartile. On a categorical scale, compared with that of the first quartile, the multivariable adjusted odds ratio (95% CI) was 0.66 (0.46–0.95) for the fourth quartile, 0.61 (0.44–0.86) for the third quartile, and 0.89 (0.67–1.17) for the second quartile. The *P* for trend test was 0.011 across the quartiles. Details are provided in Table 2.

**Table 2:** Weighted multivariable ordinal logistic regression analysis between RC levels (log-transformed) and age-stratified quartiles of the CERAD total score

RC, mmol/L	OR (95%CI)		
	**Model 1**	**Model 2**	**Model 3**
**Continuous, mmol/L**	0.72(0.56–0.93)*	0.74(0.58–0.94)*	0.77(0.61–0.96)*
***Categorical (quartiles)***			
≤0.39 mmol/L	Ref	Ref	Ref
0.39 to ≤ 0.54 mmol/L	0.87(0.67–1.13)	0.91(0.68–1.21)*	0.93(0.70–1.25)
0.54 to ≤ 0.78 mmol/L	0.56(0.40–0.80)**	0.62(0.44–0.88)**	0.64(0.47–0.88)**
> 0.78 mmol/L	0.66(0.44–0.98)*	0.67(0.46–0.98)*	0.70(0.50–0.98)*
*P for linear trend*	0.040	0.015	0.013

Model 1 univariate analysis

Model 2 adjusted for age, gender, education level, and race

Model 3 further adjusted for BMI status, smoking status, and drinking status plus model 2

^*^P < 0.05, **P < *0.01*

On a continuous scale, each 1 unit increase in the TC/RC ratio was associated with a multivariable adjusted odds ratio (95% CI) of 1.45 (1.13–1.87) for having a CERAD total score in a higher quartile. On a categorical scale, compared with that of the first quartile, the multivariable adjusted odds ratio (95% CI) was 1.50 (1.03–2.18) for the fourth quartile, 1.38 (1.07–1.78) for the third quartile, and 1.12 (0.81–1.55) for the second quartile. The *P* for trend test was 0.020 across the quartiles. Details are provided in Table 3.

**Table 3:** Weighted multivariable ordinal logistic regression analysis between the TC/RC ratio (log-transformed) and age-stratified quartiles of the CERAD total score

TC/RC	OR (95% CI)		
	**Model 1**	**Model 2**	**Model 3**
**Continuous**	1.68(1.29–2.19)**	1.46(1.12–1.90)**	1.40(1.10–1.78)**
***Categorical (quartiles)***			
≤6.08	Ref	Ref	Ref
> 6.08 to ≤8.75	1.12(0.80–1.56)	1.14(0.83–1.56)	1.09(0.79–1.51)
> 8.75 to ≤12.94	1.38(1.09–1.76)**	1.42(1.10–1.84)*	1.33(1.03–1.71)*
> 12.94	1.87(1.25–2.80)**	1.51(1.02–2.22)*	1.41(1.00–1.97)*
*P for linear trend*	0.002	0.020	0.028

Model 1 univariate analysis

Model 2 adjusted for age, gender, education level, and race

Model 3 further adjusted for BMI status, smoking status, and drinking status plus model 2

^*^P < 0.05, **P < 0.01

### Subgroup analysis

**Fig. 2:**
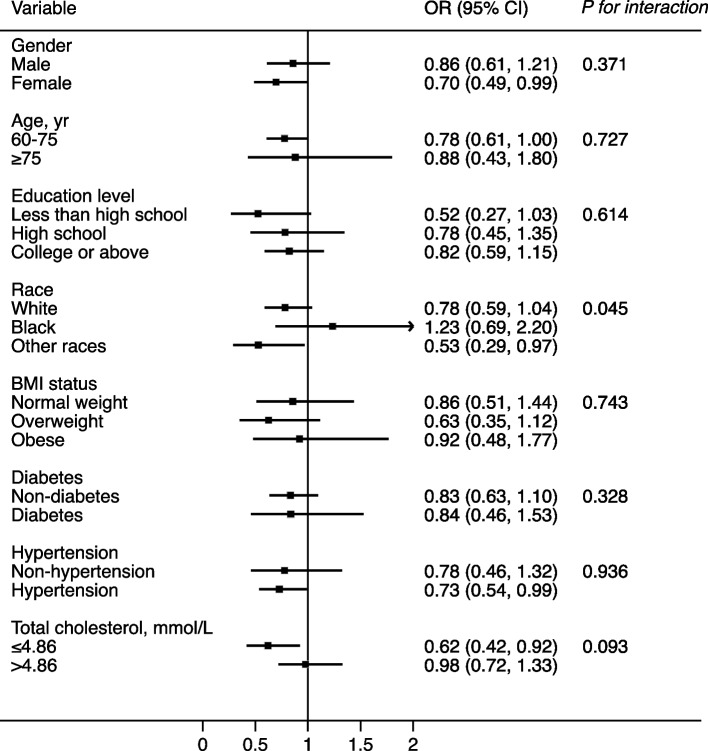
Effect size of RC levels on the age-stratified quartiles of the CERAD total score in subgroups. Notes: Model adjusted for age, sex, educational level, race, BMI status, smoking status, and drinking status

**Fig. 3:**
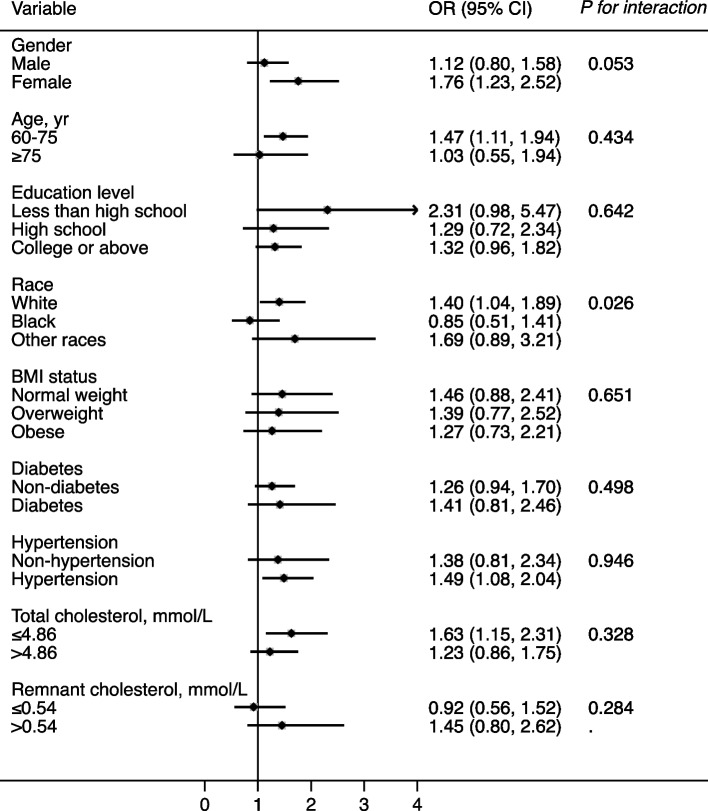
Effect size of the TC/RC ratio on the age-stratified quartiles of the CERAD total score in subgroups. Notes: Model adjusted for age, sex, educational level, race, BMI status, smoking status, and drinking status

Stratified analyses were conducted for the relationship between RC levels, the TC/RC ratio, and age-stratified quartiles of the CERAD total score (Figs. 2 and 3). After adjusting for confounders, the effect size of the RC level as well as the TC/RC ratio on quartiles of the CERAD total score were almost all consistent across the subgroups, including sex, age, education level, BMI status, diabetes, hypertension, and TC level (*P for interaction* ≥0.05). Only the race of black was an exception, which deserves further study.

## Discussion

To our knowledge, this is the first study to explore the association between plasma RC levels, as well as the TC to RC ratio, and cognitive function. The present study had the following important findings. First, a higher level of RC was associated with a higher risk of verbal learning and memory function impairment. Second, a higher TC to RC ratio was associated with a lower risk of verbal learning and memory function impairment. Third, in comparison with RC levels, the TC/RC ratio showed a steadier relationship with verbal learning and memory function under multiple analytic approaches. Fourth, the effect sizes of RC levels as well as the TC/RC ratio on verbal learning and memory function were consistent across almost all subgroup analyses.

RC is defined as the cholesterol composition of remnants that are metabolized from TG-rich lipoproteins, including chylomicron and very low-density lipoprotein (VLDL) [17]. Quite a few observational studies have investigated the relationship between classical lipid components, such as TC, LDL-C, and HDL-C, and cognitive function. However, there is no current laboratory or epidemiological evidence available concerning the association between RC and cognitive function and the underlying mechanisms. Here, for the first time, this study provides a glimpse of the relationship between RC and cognitive function, suggesting that a lower RC level is associated with better verbal learning and memory function defined by the CERAD total score. The CERAD WL subtest is part of the CERAD Neuropsychology battery, which was originally designed to permit staging of AD. Utilizing a multiple analytic approach, the study demonstrated that a higher level of RC was correlated with worse verbal learning and memory function in American elderly individuals aged ≥60 years.

Although no relevant study is currently available, the study results have biologically plausible explanations. Remnants with diameters less than 70 nm can penetrate the intima of the arterial wall, leading to atherosclerosis [18]. The relationship between RC and atherosclerotic diseases [19], as well as its association with cardiovascular outcomes independent of LDL-C [20], has been established in previous studies. Due to its atherogenic properties, RC can contribute to atherosclerosis in both the carotid artery [21] and arterioles in the brain. A Mendelian randomization study confirmed the causal relationship of remnant lipoprotein-associated genes and ischemic stroke [22]. Prospective cohort studies on symptomatic intracranial atherosclerotic stenosis and ischemic stroke indicated that RC causes cerebral hypoperfusion [23]. In addition, population-based studies showed that a reduction in cerebral perfusion was associated with an increased risk of dementia [24, 25]. Previous studies also reported that RC enhanced oxidative stress and proinflammatory effects on vascular endothelial and smooth muscle cells [26], which might damage the blood‒brain barrier, subsequently altering amyloid degradation and cholesterol homeostasis [27] in the brain. A recent Mendelian randomization study on the risk factors for AD found that genetically elevated TC and LDL-C levels increased neurotic plaque burden, but the effects were driven by single nucleotide polymorphisms of *APOE* [28], whose genetic product is known to be the key ligand for remnant lipoprotein clearance by the liver. Combined with the results of the present study, disordered APOE-mediated clearance of remnant lipoprotein might partly participate in the development of cognitive impairment. Further research should be conducted to determine the underlying mechanisms and their clinical significance.

The present study proposed a new blood lipid index, the TC/RC ratio. The adverse effect of high TC levels on cognitive function has been abundantly investigated in previous studies. However, serum TC includes cholesterol molecules from a variety of subtypes of lipoprotein, and lowering cholesterol of different lipoprotein subtypes might produce differential outcomes. Therefore, the beneficial effect of cholesterol-lowering therapy on cognitive function is still controversial, especially in elderly individuals [29]. Based on previous studies and the relationship between RC levels and the CERAD total score found in the present study, it is supposed that TC, in combination with RC, might be a better bioindex for the prediction of cognitive function than TC or RC separately. In light of this, the present study examined and demonstrated that a higher TC/RC ratio is suggestive of better verbal learning and memory function assessed by CERAD tests.

In addition, the present study found that the TC/RC ratio showed a significant positive association not only with the CERAD total score but also with the CERAD delayed trial test score [see Additional file 1, Table S1], while RC levels showed no association with delayed trial test score [see Additional file 1, Table S2]. Previous studies have found that some of the measures in the CERAD WL subtest, particularly delayed recall of a word list, could more efficiently distinguish persons with dementia from those with normal cognition [30]. Therefore, the results of the present study indicated that the TC/RC ratio might have better value for predicting verbal learning and memory function than RC levels.

### Comparisons with other studies and what the current work adds to the existing knowledge

Conclusively, the current study examined two available blood lipid indices for the assessment of verbal learning and memory function, which have not been reported by previous studies. For future clinical application, the present study provided evidence for the utilization of RC levels and the TC/RC ratio in the evaluation of verbal learning and memory function, which might assist in risk stratification for cognitive function impairment or AD susceptibility in elderly individuals. Considering both cardiovascular and cognitive benefits implied by the present study, the lower the RC level, the better. Regarding the benefit of a higher TC/RC ratio for verbal learning and memory function, there might be a potential atherogenic risk when the higher ratio is mainly attributed to a relatively high TC level rather than a low RC level. However, in the context of this study, the mean TC level was 4.97 ± 1.07 mmol/L, which was not a significant atherogenic level.

### Study strengths and limitations

There are several strengths in the present study. First, the study observed a known cardiovascular risk factor, RC, and its negative relationship with verbal learning and memory function. Moreover, the study utilized data extracted from the NHANES database, which used complex, multistage sampling, and relatively convincing results could be drawn with a proper analytic approach. Additionally, the study proposed an RC derivative, the TC/RC ratio, and found its positive association with verbal learning and memory function, which had not been mentioned previously. Additionally, the results were estimated in several subpopulations, verifying its authenticity.

The study has several limitations. First, this was an observational study with a cross-sectional design, and the causal relationship could not be determined between RC levels or the TC/RC ratio and cognitive function. Second, in this study, LDL-C was calculated based on the Friedewald equation, which is not applicable when TG levels > 400 mg/dl. Therefore, the results of the study should not be interpreted in the situation of very high TG levels. Third, the present study used calculated fasting RC to represent the “remnant” cholesterol level; however, this calculated fasting RC includes not only cholesterol from remnants but also cholesterol from newly formed VLDL particles, which in fact overestimates the cholesterol levels of actual remnants. However, RC from the indirect formula has been widely used in numerous clinical studies and has been proven to be a convenient and reliable risk predictor. Finally, although the present study included many important potential covariates previously reported to affect cognition in the statistical models, the possibility that residual confounding factors remain could not be ruled out. Future large-scale, prespecified trials are needed to further explore this subject.

## Conclusions

Utilizing a cohort of American elderly individuals aged over 60 years from the NHANES database, the present study found that in the context of a TG level below 400 mg/dl, a lower RC level and a higher TC/RC ratio were associated with better verbal learning and memory function. The present study indicated that lowering RC levels or increasing the TC/RC ratio when the TG level is below 400 mg/dl could possibly be beneficial for preventing cognitive impairment in elderly individuals. The study results, which need to be verified in future larger cohort studies, might help in guiding risk prediction and primary prevention of cognitive impairment in elderly individuals.

## Supplementary Information

**Additional file 1: Table S1.** Weighted multivariable ordinal logistic regression analysis between RC levels (log-transformed) and age-stratified quartiles of the CERAD delayed score. **Table S2.** Weighted multivariable ordinal logistic regression analysis between the TC/RC ratio (log-transformed) and age-stratified quartiles of the CERAD delayed score.

## Data Availability

The datasets analyzed during the current study are available on the NHANES official website, https://wwwn.cdc.gov/Nchs/Nhanes/.

## Abbreviations

RC: Remnant cholesterol
TC/RC: Total cholesterol/remnant cholesterol
CERAD: Consortium to Establish a Registry for Alzheimer’s Disease Word List Memory Task
NHANES: National Health and Nutrition Examination Survey
TC: Total cholesterol
LDL: Low-density lipoprotein
HDL: High-density lipoprotein
LDL-C: Low-density lipoprotein cholesterol
HDL-C: High-density lipoprotein cholesterol
TG: Triglycerides
VLDL: Very low-density lipoprotein
BMI: Body mass index
