# Supplementary material for: Association between remnant cholesterol and verbal learning and memory function in the elderly in the US
Source: Lipids Health Dis. 2022 Nov 14;21:120. doi: 10.1186/s12944-022-01729-4 (PMC9664689; doi:10.1186/s12944-022-01729-4)
Supplement: Supplementary file 1 — Additional file 1: Table S1. Weighted multivariable ordinal logistic regression analysis between RC levels (log-transformed) and age-stratified quartiles of the CERAD delayed score. Table S2. Weighted multivariable ordinal logistic regression analysis between the TC/RC ratio (log-transformed) and age-stratified quartiles of the CERAD delayed score. [file 12944_2022_1729_MOESM1_ESM.docx]

**Table S1 Weighted multivariable ordinal logistic regression analysis between RC levels (log-transformed) and age-stratified quartiles of the CERAD delayed score.**

| RC, mmol/L | OR (95%CI) | | |
| --- | --- | --- | --- |
|  | **Model 1** | **Model 2** | **Model 3** |
| **Continuous, mmol/L** | 0.79(0.61,1.03) | 0.82(0.63,1.06) | 0.82(0.64,1.04) |
| ***Categorical (quartiles)*** | | | |
| $\leq$0.39mmol/L | Ref. | Ref. | Ref. |
| 0.39 to $\leq$ 0.54mmol/L | 0.82(0.62,1.10) | 0.87(0.65,1.16) | 0.86(0.65,1.14) |
| 0.54 to $\leq$ 0.78mmol/L | 0.68(0.48,0.95)* | 0.75(0.55,1.04) | 0.75(0.55,1.01) |
| >0.78mmol/L | 0.65(0.43,1.00)* | 0.67(0.44,1.02) | 0.67(0.44,1.02) |
| **Model 1** univariate analysis; **Model 2** adjusted for age, sex, education level, and race; **Model 3** further adjusted for smoking status and drinking status plus model 2. **P<0.05, **P<0.01* | | | |

**Table S2 Weighted multivariable ordinal logistic regression analysis between the TC/RC ratio (log-transformed) and age-stratified quartiles of the CERAD delayed score.**

| TC/RC | OR (95% CI) | | |  |
| --- | --- | --- | --- | --- |
|  | **Model 1** | **Model 2** | **Model 3** |  |
| **Continuous** | 1.51(1.16,1.95)** | 1.35(1.03,1.76)* | 1.34(1.04,1.72)* |  |
| ***Categorical (quartiles)*** | | | |  |
| $\leq$6.08 | Ref. | Ref. | Ref. |  |
| >6.08 to $\leq$8.75 | 1.34(0.97,1.84) | 1.41(0.99,2) | 1.35(0.94,1.94) |  |
| >8.75 to $\leq$12.94 | 1.44(1.1,1.89)** | 1.52(1.14,2.04)** | 1.44(1.09,1.9)* |  |
| >12.94 | 1.75(1.16,2.62)** | 1.48(0.98,2.25) | 1.46(0.98,2.17) |  |
| **Model 1** univariate analysis; **Model 2** adjusted for age, sex, education level, and race; **Model 3** further adjusted for smoking status and drinking status plus model 2. **P<0.05, **P<0.01* | | | | |
